# 1,4-Bis(imidazol-1-yl)benzene–terephthalic acid (1/1)

**DOI:** 10.1107/S1600536808040324

**Published:** 2008-12-06

**Authors:** Shiyong Zhang, Yurong Tang, Zhihua Mao, Mingliang Li, Jingbo Lan, Xiaoyu Su

**Affiliations:** a College of Chemistry, Sichuan University, Chengdu 610064, People’s Republic of China; b The Center of Testing and Analysis, Sichuan University, Chengdu 610064, People’s Republic of China

## Abstract

In the title compound, C_12_H_10_N_4_·C_8_H_6_O_4_, 1,4-bis­(imidazol-1-yl)benzene and terephthalic acid mol­ecules are joined *via* strong O—H⋯N hydrogen bonds to form infinite zigzag chains. Both mol­ecules are located on crystallographic inversion centers. The O—H⋯N hydrogen-bonded chains are assembled into two-dimensional layers through weak C—H⋯O and strong π–π stacking inter­actions [centroid–centroid distance = 3.818 (2) Å], leading to the formation of a three-dimensional supra­molecular structure.

## Related literature

For general background, see: Aakeröy *et al.* (2006 ▶); Aakeröy & Seddon (1993 ▶); Desiraju, 2007 ▶; Corna *et al.* (2004 ▶); Dobrzanska *et al.* (2006); Van Roey *et al.* (1991 ▶). For similar structures, see: Wang *et al.* (2007 ▶); Su *et al.* (2007 ▶).
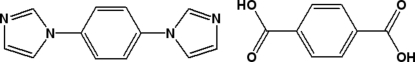


## Experimental

### 

#### Crystal data

C_12_H_10_N_4_·C_8_H_6_O_4_
*M* *_r_* = 376.37
Monoclinic, 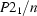

*a* = 5.2780 (17) Å
*b* = 10.599 (5) Å
*c* = 15.449 (5) Å
β = 91.17 (3)°
*V* = 864.1 (6) Å^3^
*Z* = 2
Mo *K*α radiation
μ = 0.10 mm^−1^
*T* = 293 (2) K
0.25 × 0.22 × 0.15 mm

#### Data collection

Enraf–Nonius CAD-4 diffractometer
Absorption correction: none
1895 measured reflections
1538 independent reflections
904 reflections with *I* > 2σ(*I*)
*R*_int_ = 0.008
3 standard reflections every 200 reflections intensity decay: 2.5%

#### Refinement

*R*[*F* ^2^ > 2σ(*F* ^2^)] = 0.045
*wR*(*F* ^2^) = 0.132
*S* = 0.95
1538 reflections
128 parameters
H-atom parameters constrained
Δρ_max_ = 0.18 e Å^−3^
Δρ_min_ = −0.21 e Å^−3^

### 

Data collection: *DIFRAC* (Gabe & White, 1993 ▶); cell refinement: *DIFRAC*; data reduction: *NRCVAX* (Gabe *et al.*, 1989 ▶); program(s) used to solve structure: *SHELXS97* (Sheldrick, 2008 ▶); program(s) used to refine structure: *SHELXL97* (Sheldrick, 2008 ▶); molecular graphics: *ORTEPIII* (Burnett & Johnson, 1996 ▶) and *Mercury* (Macrae *et al.*, 2006 ▶); software used to prepare material for publication: *SHELXL97*.

## Supplementary Material

Crystal structure: contains datablocks global, I. DOI: 10.1107/S1600536808040324/zl2155sup1.cif

Structure factors: contains datablocks I. DOI: 10.1107/S1600536808040324/zl2155Isup2.hkl

Additional supplementary materials:  crystallographic information; 3D view; checkCIF report

## Figures and Tables

**Table 1:** Hydrogen-bond geometry (Å, °)

*D*—H⋯*A*	*D*—H	H⋯*A*	*D*⋯*A*	*D*—H⋯*A*
O1—H1⋯N2^i^	0.82	1.79	2.60885	178
C2—H2⋯O2^ii^	0.93	2.54	3.376 (3)	150
C4—H4⋯O2^iii^	0.93	2.56	3.463 (3)	162

Symmetry codes: (i) 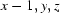; (ii) 
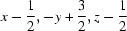
; (iii) 
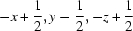
.

**Fig. 1.**
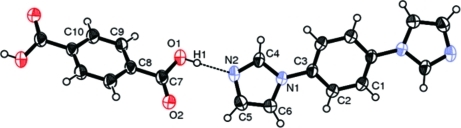
The molecular structure of the title compound, showing 50% probability displacement ellipisoids; hydrogen bonds are illustrated by dashed lines.

**Fig. 2.**
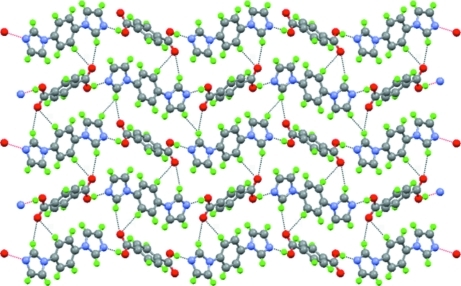
A two-dimensional network layer of the title compound viewed along the c axis. Dashed lines indicate O—H···N and C—H···O hydrogen bonds. C, gray; H, green; O, red; N, cyan.

**Fig. 3.**
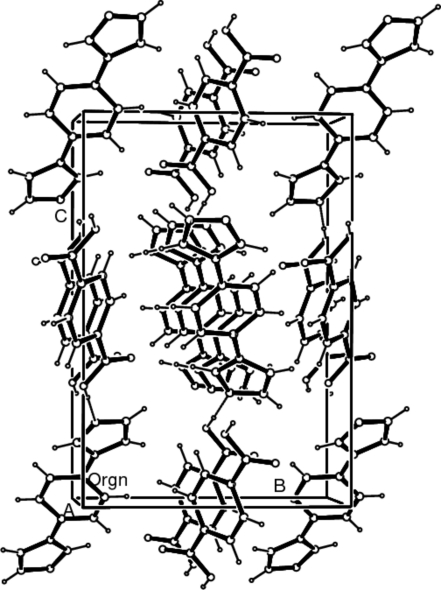
A three-dimensional view of the supramolecular layers of the title compound.

## supplementary crystallographic information

### Comment

Supramolecular architectures assembled via various delicate noncovalent interactions such as hydrogen bonds, π—π stacking and electrostatic interactions, *etc*., have attracted intense interest in recent years because of their fascinating structural diversity and potential applications for functional materials (Desiraju, 2007; Corna *et al.*, 2004). Especially, the application of intermolecular hydrogen bonds is a well known and efficient tool in the field of organic crystal design owing to its strength and directional properties (Aakeröy & Seddon, 1993). Imidazoles, as excellent N-donor compounds, have attracted special attention in the construction of organic cocrystals in recent years (Aakeröy *et al.*, 2006; Van Roey *et al.*, 1991). We recently presented organic crystals composed of flexible diimidazole compounds and dicarboxylic acids (Wang *et al.*, 2007; Su *et al.*, 2007). In further development of such interesting hydrogen-bonded supramolecular systems and as a continuation of our research in this area, we report herein the crystal structure of the title compound formed from rigid diimidazole and dicarboxylic acids molecules.

As shown in Figure 1, the asymmetric unit of the title compound contains each half a molecule of 1,4-bis(1-imidazolyl)benzene and terephthalic acid which are both located on crystallographic inversion centers. The carboxyl groups of the terephthalic acid interact with the imidazol-1-yl nitrogen atoms of 1,4-bis(1-imidazolyl)benzene *via* O1—H1···N2 hydrogen bonds (O1···N2 = 2.608 (8) Å and O1—H1···N2 = 178°), and thus the hydrogen bonds further propagate the acid-base subunits into an infinite one-dimensional zig-zag chain. Meanwhile, these chains are assembled into two-dimensional layers through weak C2—H2···O2 and C4—H4···O2 hydrogen bonds, with C2···O2 = 3.376 (3) Å, C2—H2···O2 = 150° and C4···O2 = 3.463 (3) Å, C4—H4···O2 = 162° (Figure 2). Moreover, the supramolecular layers are further stablized by intermolecular π—π interactions to form a three-dimensional stucture as depicted in Figure 3. A relative strong π—π interaction between one imidazole ring (*Cg*1: N1—C4—N2—C5—C6) and contiguous phenyl ring (*Cg*2: C1—C3—C2—C1b—C3b—C2b) of 1,4-bis(1-imidazolyl)benzene from another chain (centroid-centroid distance = 3.818 (2) Å, dihedral angle = 27.12) plays an important part in the connection of adjacent layers, where *Cg*1 is the centroid of the imidazole ring of the molecule at (1 - *x*, 1 - *y*, -*z*) and (-1 + *x*, *y*, *z*), and *Cg*2 is the centroid of the phenyl ring of the molecule at (1 - *x*, 1 - *y*, -*z*) and (1 + *x*, *y*, *z*).

### Experimental

A methanol solution (5 ml) of 1,4-bis(1-imidazolyl)benzene (0.05 mmol, 10.5 mg) was added slowly with constant stirring to a solution of terephthalic acid (0.05 mmol, 8.3 mg) in methanol (2 ml) and water (0.5 ml) to give a clear solution. Then the reaction mixture was filtered and left to stand at room temperature. Colorless block crystals suitable for X-ray analysis were obtained after one week by slow evaporation of the solvent. Yield, 85%; ^1^H NMR (400 MHz, DMSO-d6): δ 8.34 (s, 2H), 8.04 (s, 4H), 7.83 (t, 6H), 7.13 (s, 2H).

### Refinement

All non-hydrogen atoms were located using direct methods and successive difference Fourier syntheses, and refined with anisotropic thermal parameters. All hydrogen atoms were positioned theoretically and refined with the riding model approximation with C—H = 0.93–0.97 Å and *U*_iso_(H) = 1.2*U*_eq_(C), and O—H = 0.82 Å and *U*_iso_(H) = 1.5*U*_eq_(O)

### Crystal data

**Table:**

C_12_H_10_N_4_·C_8_H_6_O_4_	*F*(000) = 392
*M**_r_* = 376.37	*D*_x_ = 1.447 Mg m^−^^3^
Monoclinic, *P*2_1_/*n*	Mo *K*α radiation, λ = 0.71073 Å
Hall symbol: -P 2yn	Cell parameters from 18 reflections
*a* = 5.2780 (17) Å	θ = 4.5–7.6°
*b* = 10.599 (5) Å	µ = 0.10 mm^−^^1^
*c* = 15.449 (5) Å	*T* = 293 K
β = 91.17 (3)°	Block, colourless
*V* = 864.1 (6) Å^3^	0.25 × 0.22 × 0.15 mm
*Z* = 2	

### Data collection

**Table:**

Enraf–Nonius CAD-4 diffractometer	*R*_int_ = 0.008
Radiation source: fine-focus sealed tube	θ_max_ = 25.5°, θ_min_ = 2.3°
graphite	*h* = −6→6
ω/2θ scans	*k* = 0→12
1895 measured reflections	*l* = −9→18
1538 independent reflections	3 standard reflections every 200 reflections
904 reflections with *I* > 2σ(*I*)	intensity decay: 2.5%

### Refinement

**Table:**

Refinement on *F*^2^	Primary atom site location: structure-invariant direct methods
Least-squares matrix: full	Secondary atom site location: difference Fourier map
*R*[*F*^2^ > 2σ(*F*^2^)] = 0.045	Hydrogen site location: inferred from neighbouring sites
*wR*(*F*^2^) = 0.132	H-atom parameters constrained
*S* = 0.95	*w* = 1/[σ^2^(*F*_o_^2^) + (0.0842*P*)^2^] where *P* = (*F*_o_^2^ + 2*F*_c_^2^)/3
1538 reflections	(Δ/σ)_max_ < 0.001
128 parameters	Δρ_max_ = 0.18 e Å^−^^3^
0 restraints	Δρ_min_ = −0.21 e Å^−^^3^

### Special details

**Table:**

Experimental. Refinement of F^2^ against ALL reflections. The weighted *R*-factor *wR* and goodness of fit S are based on F^2^, conventional *R*-factors *R* are based on F, with F set to zero for negative F^2^. The threshold expression of F^2^ > 2sigma(F^2^) is used only for calculating *R*-factors(gt) *etc*. and is not relevant to the choice of reflections for refinement. *R*-factors based on F^2^ are statistically about twice as large as those based on F, and R– factors based on ALL data will be even larger.
Geometry. All e.s.d.'s (except the e.s.d. in the dihedral angle between two l.s. planes) are estimated using the full covariance matrix. The cell e.s.d.'s are taken into account individually in the estimation of e.s.d.'s in distances, angles and torsion angles; correlations between e.s.d.'s in cell parameters are only used when they are defined by crystal symmetry. An approximate (isotropic) treatment of cell e.s.d.'s is used for estimating e.s.d.'s involving l.s. planes.
Refinement. Refinement of *F*^2^ against ALL reflections. The weighted *R*-factor *wR* and goodness of fit *S* are based on *F*^2^, conventional *R*-factors *R* are based on *F*, with *F* set to zero for negative *F*^2^. The threshold expression of *F*^2^ > σ(*F*^2^) is used only for calculating *R*-factors(gt) *etc*. and is not relevant to the choice of reflections for refinement. *R*-factors based on *F*^2^ are statistically about twice as large as those based on *F*, and *R*- factors based on ALL data will be even larger.

### Fractional atomic coordinates and isotropic or equivalent isotropic displacement parameters (Å^2^)

**Table:**

	*x*	*y*	*z*	*U*_iso_*/*U*_eq_	
N1	0.3750 (4)	0.57026 (17)	0.12218 (11)	0.0403 (5)	
N2	0.7031 (4)	0.5588 (2)	0.21140 (12)	0.0489 (6)	
C1	0.0819 (5)	0.6228 (2)	0.00451 (16)	0.0467 (6)	
H1A	0.1382	0.7059	0.0073	0.056*	
C2	−0.1041 (5)	0.5891 (2)	−0.05539 (15)	0.0467 (6)	
H2	−0.1745	0.6495	−0.0923	0.056*	
C3	0.1844 (4)	0.5339 (2)	0.06017 (13)	0.0375 (5)	
C4	0.5583 (4)	0.4962 (2)	0.15707 (14)	0.0445 (6)	
H4	0.5788	0.4112	0.1440	0.053*	
C5	0.6087 (5)	0.6786 (2)	0.21254 (16)	0.0531 (7)	
H5	0.6740	0.7444	0.2460	0.064*	
C6	0.4073 (3)	0.68757 (13)	0.15820 (8)	0.0504 (7)	
H6	0.3095	0.7590	0.1472	0.061*	
O1	0.0950 (3)	0.49725 (13)	0.30802 (8)	0.0499 (5)	
H1	−0.0288	0.5179	0.2784	0.075*	
O2	0.0072 (4)	0.67524 (16)	0.37876 (12)	0.0601 (6)	
C7	0.1260 (5)	0.5775 (2)	0.37186 (15)	0.0428 (6)	
C8	0.3231 (4)	0.5370 (2)	0.43721 (14)	0.0387 (6)	
C9	0.5136 (4)	0.4536 (2)	0.41709 (14)	0.0415 (6)	
H9	0.5236	0.4221	0.3611	0.050*	
C10	0.6897 (4)	0.4164 (2)	0.47922 (14)	0.0435 (6)	
H10	0.8171	0.3600	0.4650	0.052*	

### Atomic displacement parameters (Å^2^)

**Table:**

	*U*^11^	*U*^22^	*U*^33^	*U*^12^	*U*^13^	*U*^23^
N1	0.0420 (11)	0.0393 (11)	0.0391 (10)	0.0033 (9)	−0.0132 (9)	0.0005 (8)
N2	0.0476 (12)	0.0568 (13)	0.0415 (11)	−0.0001 (11)	−0.0184 (9)	−0.0007 (9)
C1	0.0516 (15)	0.0374 (12)	0.0504 (13)	0.0036 (11)	−0.0187 (12)	0.0008 (10)
C2	0.0542 (15)	0.0390 (13)	0.0460 (12)	0.0053 (12)	−0.0196 (11)	0.0056 (10)
C3	0.0369 (12)	0.0418 (13)	0.0334 (11)	0.0059 (10)	−0.0102 (9)	−0.0028 (9)
C4	0.0446 (13)	0.0476 (14)	0.0405 (12)	0.0064 (12)	−0.0176 (10)	−0.0021 (10)
C5	0.0619 (16)	0.0460 (14)	0.0506 (14)	−0.0053 (13)	−0.0198 (12)	−0.0023 (12)
C6	0.0588 (16)	0.0377 (14)	0.0539 (15)	0.0035 (12)	−0.0198 (13)	−0.0039 (11)
O1	0.0495 (10)	0.0531 (10)	0.0462 (9)	0.0021 (8)	−0.0205 (7)	−0.0019 (8)
O2	0.0645 (12)	0.0425 (10)	0.0720 (13)	0.0053 (9)	−0.0335 (10)	−0.0041 (9)
C7	0.0400 (13)	0.0422 (14)	0.0457 (13)	−0.0084 (12)	−0.0128 (11)	0.0030 (11)
C8	0.0412 (13)	0.0335 (12)	0.0409 (12)	−0.0106 (10)	−0.0133 (10)	0.0058 (9)
C9	0.0427 (13)	0.0441 (13)	0.0372 (11)	−0.0040 (11)	−0.0087 (10)	−0.0003 (10)
C10	0.0363 (13)	0.0440 (14)	0.0498 (13)	−0.0044 (11)	−0.0083 (11)	0.0019 (11)

### Geometric parameters (Å, °)

**Table:**

N1—C4	1.349 (3)	C5—H5	0.9300
N1—C6	1.371 (2)	C6—H6	0.9300
N1—C3	1.428 (3)	O1—C7	1.31010
N2—C4	1.305 (3)	O1—H1	0.8200
N2—C5	1.364 (3)	O2—C7	1.217 (3)
C1—C3	1.379 (3)	C7—C8	1.498 (3)
C1—C2	1.383 (3)	C8—C9	1.379 (3)
C1—H1A	0.9300	C8—C10^ii^	1.385 (3)
C2—C3^i^	1.372 (3)	C9—C10	1.380 (3)
C2—H2	0.9300	C9—H9	0.9300
C3—C2^i^	1.372 (3)	C10—C8^ii^	1.385 (3)
C4—H4	0.9300	C10—H10	0.9300
C5—C6	1.344 (3)		
			
C4—N1—C6	106.45 (17)	N2—C5—H5	125.0
C4—N1—C3	126.9 (2)	C5—C6—N1	106.22 (16)
C6—N1—C3	126.66 (17)	C5—C6—H6	126.9
C4—N2—C5	105.8 (2)	N1—C6—H6	126.9
C3—C1—C2	120.3 (2)	C7—O1—H1	109.5
C3—C1—H1A	119.8	O2—C7—O1	124.20
C2—C1—H1A	119.8	O2—C7—C8	122.5 (2)
C3^i^—C2—C1	119.7 (2)	O1—C7—C8	113.31
C3^i^—C2—H2	120.1	C9—C8—C10^ii^	119.2 (2)
C1—C2—H2	120.1	C9—C8—C7	122.1 (2)
C2^i^—C3—C1	119.9 (2)	C10^ii^—C8—C7	118.7 (2)
C2^i^—C3—N1	120.35 (19)	C8—C9—C10	120.7 (2)
C1—C3—N1	119.7 (2)	C8—C9—H9	119.7
N2—C4—N1	111.5 (2)	C10—C9—H9	119.7
N2—C4—H4	124.2	C9—C10—C8^ii^	120.1 (2)
N1—C4—H4	124.2	C9—C10—H10	119.9
C6—C5—N2	110.01 (19)	C8^ii^—C10—H10	119.9
C6—C5—H5	125.0		
			
C3—C1—C2—C3^i^	−0.9 (4)	N2—C5—C6—N1	0.0 (3)
C2—C1—C3—C2^i^	0.9 (4)	C4—N1—C6—C5	0.3 (2)
C2—C1—C3—N1	−179.5 (2)	C3—N1—C6—C5	−179.9 (2)
C4—N1—C3—C2^i^	26.8 (4)	O2—C7—C8—C9	−157.3 (2)
C6—N1—C3—C2^i^	−153.0 (2)	O1—C7—C8—C9	23.07
C4—N1—C3—C1	−152.9 (2)	O2—C7—C8—C10^ii^	23.6 (4)
C6—N1—C3—C1	27.3 (3)	O1—C7—C8—C10^ii^	−155.90
C5—N2—C4—N1	0.5 (3)	C10^ii^—C8—C9—C10	0.2 (4)
C6—N1—C4—N2	−0.5 (3)	C7—C8—C9—C10	−178.8 (2)
C3—N1—C4—N2	179.6 (2)	C8—C9—C10—C8^ii^	−0.2 (4)
C4—N2—C5—C6	−0.3 (3)		

Symmetry codes: (i) −*x*, −*y*+1, −*z*; (ii) −*x*+1, −*y*+1, −*z*+1.

### Hydrogen-bond geometry (Å, °)

**Table:**

*D*—H···*A*	*D*—H	H···*A*	*D*···*A*	*D*—H···*A*
O1—H1···N2^iii^	0.82	1.79	2.60885	178
C2—H2···O2^iv^	0.93	2.54	3.376 (3)	150
C4—H4···O2^v^	0.93	2.56	3.463 (3)	162

Symmetry codes: (iii) *x*−1, *y*, *z*; (iv) *x*−1/2, −*y*+3/2, *z*−1/2; (v) −*x*+1/2, *y*−1/2, −*z*+1/2.
